# A Python library for FAIRer access and deposition to the Metabolomics Workbench Data Repository

**DOI:** 10.1007/s11306-018-1356-6

**Published:** 2018-04-20

**Authors:** Andrey Smelter, Hunter N. B. Moseley

**Affiliations:** 1 Department of Molecular and Cellular Biochemistry, University of Kentucky, Lexington, KY 40356 USA; 2 Markey Cancer Center, University of Kentucky, Lexington, KY 40356 USA; 3 Center for Environmental and Systems Biochemistry, University of Kentucky, Lexington, KY 40356 USA; 4 Institute for Biomedical Informatics, University of Kentucky, Lexington, KY 40356 USA

**Keywords:** mwTab, Metabolomics Workbench, mwtab Python package, Data validation, FAIR

## Abstract

**Introduction:**

The Metabolomics Workbench Data Repository is a public repository of mass spectrometry and nuclear magnetic resonance data and metadata derived from a wide variety of metabolomics studies. The data and metadata for each study is deposited, stored, and accessed via files in the domain-specific ‘mwTab’ flat file format.

**Objectives:**

In order to improve the accessibility, reusability, and interoperability of the data and metadata stored in ‘mwTab’ formatted files, we implemented a Python library and package. This Python package, named ‘mwtab’, is a parser for the domain-specific ‘mwTab’ flat file format, which provides facilities for reading, accessing, and writing ‘mwTab’ formatted files. Furthermore, the package provides facilities to validate both the format and required metadata elements of a given ‘mwTab’ formatted file.

**Methods:**

In order to develop the ‘mwtab’ package we used the official ‘mwTab’ format specification. We used Git version control along with Python unit-testing framework as well as continuous integration service to run those tests on multiple versions of Python. Package documentation was developed using sphinx documentation generator.

**Results:**

The ‘mwtab’ package provides both Python programmatic library interfaces and command-line interfaces for reading, writing, and validating ‘mwTab’ formatted files. Data and associated metadata are stored within Python dictionary- and list-based data structures, enabling straightforward, ‘pythonic’ access and manipulation of data and metadata. Also, the package provides facilities to convert ‘mwTab’ files into a JSON formatted equivalent, enabling easy reusability of the data by all modern programming languages that implement JSON parsers. The ‘mwtab’ package implements its metadata validation functionality based on a pre-defined JSON schema that can be easily specialized for specific types of metabolomics studies. The library also provides a command-line interface for interconversion between ‘mwTab’ and JSONized formats in raw text and a variety of compressed binary file formats.

**Conclusions:**

The ‘mwtab’ package is an easy-to-use Python package that provides FAIRer utilization of the Metabolomics Workbench Data Repository. The source code is freely available on GitHub and via the Python Package Index. Documentation includes a ‘User Guide’, ‘Tutorial’, and ‘API Reference’. The GitHub repository also provides ‘mwtab’ package unit-tests via a continuous integration service.

**Electronic supplementary material:**

The online version of this article (10.1007/s11306-018-1356-6) contains supplementary material, which is available to authorized users.

## Introduction

The Metabolomics Workbench Data Repository is a publicly available resource for metabolomics experimental data collected from mass spectrometry (MS) and nuclear magnetic resonance (NMR) analytical platforms and associated metadata describing sample and analytical details as well as experimental design (Sud et al. 2016). Study-specific experimental data and metadata can be accessed via metabolomics workbench in the form of ‘mwTab’ formatted files as well as through a representational state transfer (REST) interface. The repository currently makes available over 630 individual ‘mwTab’ files from MS- and NMR-based studies, each file having an associated study id (non-unique identifier) and analysis id (unique identifier). The metabolomics workbench provides an official data format specification (“mwTab format specification.”—Available at: http://www.metabolomicsworkbench.org/data/tutorials.php) for the ‘mwTab’ format, which consists of sequentially ordered blocks (sections) of text data. Some of the blocks consist of data represented by ‘single key to single value’ relationships that store single pieces of information. Other blocks consist of multiple ‘tab’-separated values via ‘single key to multiple values’ or ‘multiple keys to multiple values’ relationships that store multiple pieces of information in an organized manner analogous to a relational table.

Using the Python programming language, we implemented a software package and library called ‘mwtab’ in order to improve the accessibility, interoperability, and reusability (FAIR data principles) (Wilkinson et al. 2016) of the experimental data and metadata stored in the ‘mwTab’ formatted files. The FAIR data principles, “To be findable, accessible, interoperable, and reusable”, are guiding principles for good data management and stewardship of repositories (Wilkinson et al. 2016). Python was chosen because it is an open-source programming language that runs on all major operating systems (Python Software Foundation 2013; Van Rossum and Drake 2010) and has become very popular for scientific programming (Oliphant 2007). The ‘mwtab’ package parses ‘mwTab’ formatted files into Python dictionary- and list-based data structures in order to provide ‘pythonic’ data access and manipulation interfaces within Python programs (scripts, packages, etc.). Moreover, these data structures are written in such a way that they are easily serializable into Javascript object notation (JSON) formatted files, a language-independent open-standard format used for data interchange on the web. The advantage of this Python dictionary/list/JSON data structures representation is that it simultaneously facilitates data access and manipulation of ‘mwTab’ formatted files using Python or any other programming language that implements JSON parsers (i.e. all modern programming languages). In addition to improving data accessibility, the ‘mwtab’ package provides data validation facilities, i.e. data and metadata can be validated using constraints in the form of a pre-defined schema. Validation can test a variety of conditions like specifying what types of values are possible, which keys and associated values are required, which keys and associated values are optional, the order that specific data blocks must follow, and checking for consistencies within and between files.

## Methods

### Overview of the mwTab format

**Fig. 1:**
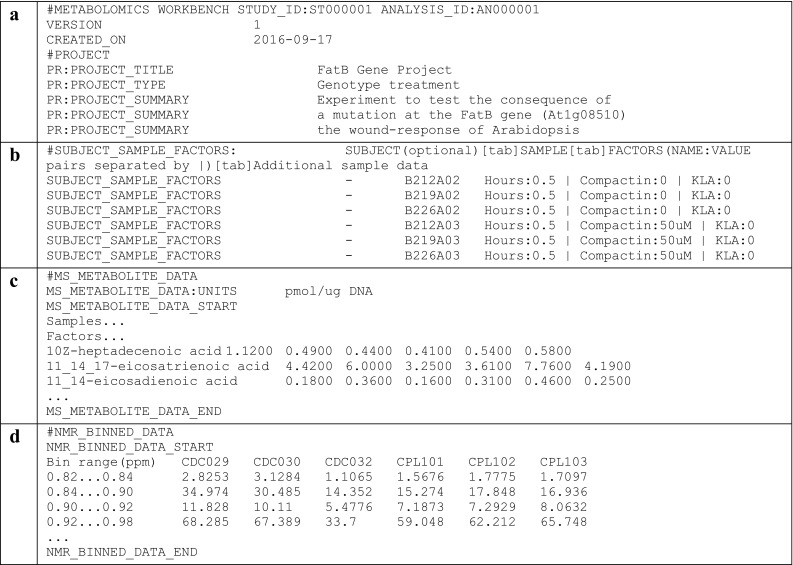
Overview of the ‘mwTab’ format: **a** Text blocks containing “single key-single value” and multiline summary blocks; **b** subject sample factors text block; **c** text block with MS metabolite data; **d** text block with NMR data

The ‘mwTab’ formatted files consists of multiple blocks of text data. Each new text block of the ‘mwTab’ file starts with the ‘#’. There are several types of formatting possible within text blocks: “single key to single value”-like pairs to represent single piece of information, e.g. ‘VERSION’ is the key and ‘1’ is the value (see Fig. 1a). In cases where value is long, it gets formatted as a multiline string with repeated use of the same key, e.g. ‘PR:PROJECT_SUMMARY’ is the key and associated multiline project summary is the value (see Fig. 1a). There is also a ‘SUBJECT_SAMPLE_FACTORS’ block that contains header specifying column names and corresponding ‘tab’-separated rows of data (see Fig. 1b). Results from MS- and NMR-based experiments are deposited as large matrices of values with corresponding units for each of the assignable metabolites (see Fig. 1c, d respectively).

The full ‘mwTab’ format specification is available on official Metabolomics Workbench Data Repository (mwTab format specification. [Online]. Available: http://www.metabolomicsworkbench.org/data/tutorials.php).

### Package implementation

**Fig. 2:**
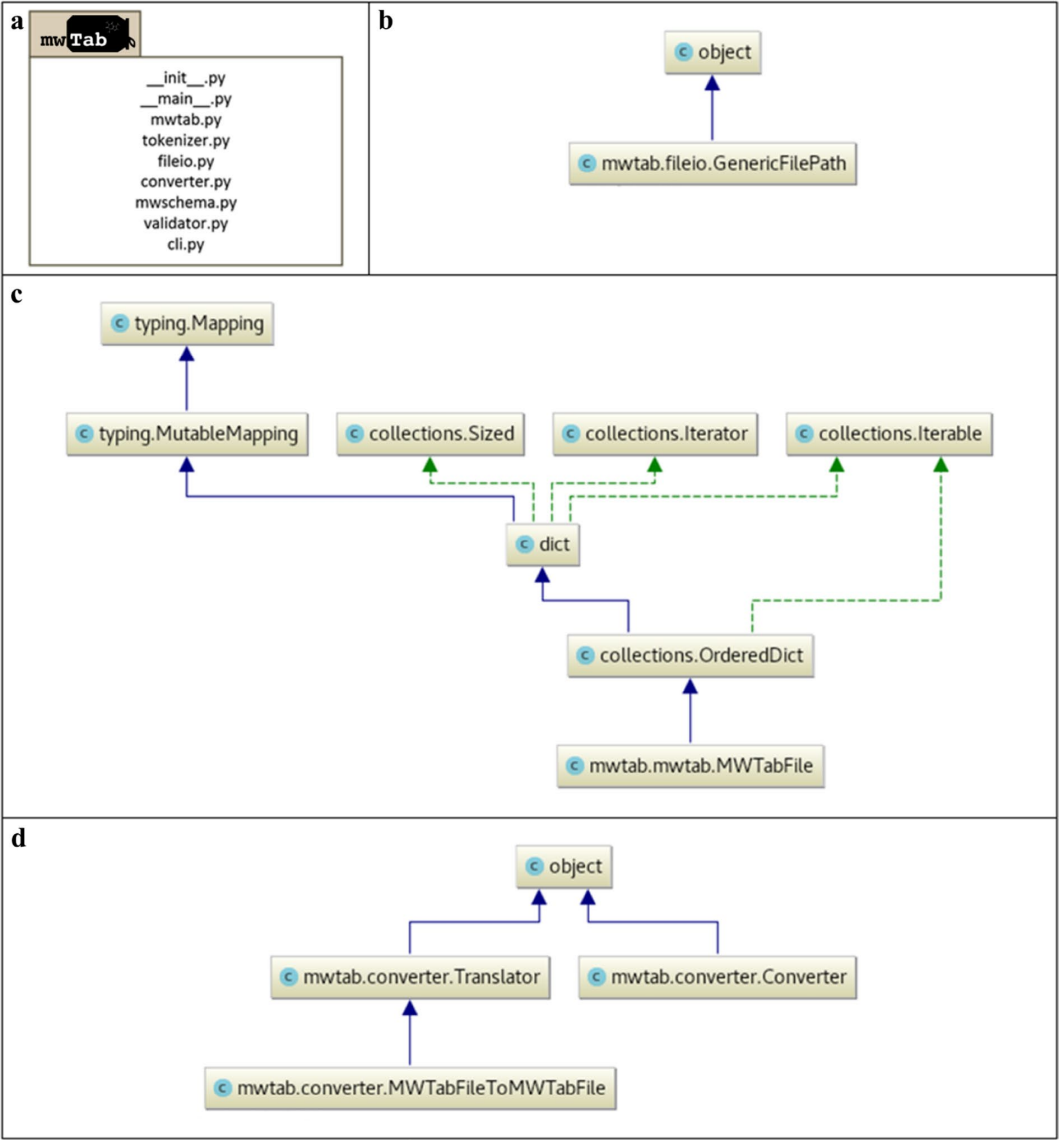
Organization of the ‘mwtab’ Python package represented with unified modeling language (UML) diagrams: **a** UML package diagram of the ‘mwtab’ Python library; **b** UML class diagram of the ‘fileio.py’ module; **c** UML class diagram of the ‘mwtab.py’ module; **d** UML class diagram of the ‘converter.py’ module

The ‘mwtab’ Python package consists of several modules: ‘mwtab.py’, ‘tokenizer.py’, ‘fileio.py’, ‘converter.py’, ‘mwschema.py’, ‘validator.py’ and ‘cli.py’ (see Fig. 2). The ‘mwtab.py’ module (Fig. 2b) implements the ‘MWTabFile’ class which can construct itself into a Python nested dictionary- and list-based data structures representation from a provided file in ‘mwTab’ format. The ‘MWTabFile’ class is the main class that provides the interfaces for data and metadata access and manipulation. The dictionary-based data structures provide key-based bracket accessors (i.e., ‘[]’) and the list-based data structures provide index-based bracket accessors (i.e., 0, 1, 2, etc.). This makes the ‘mwtab’ package a useful general-purpose library with intuitive (‘pythonic’) data access and manipulation functionality that can be integrated into higher level Python software used for downstream data analysis. The ‘tokenizer.py’ module is responsible for tokenization (lexical analysis) of the text in ‘mwTab’ format, i.e. it splits the raw text into tokens and passes them to the ‘mwtab.py’ module. Next, the ‘mwtab.py’ analyzes the tokens (syntactic analysis) and reformats them into a ‘MWTabFile’ instance with Python dictionary- and list-based instances (objects). The ‘fileio.py’ module (see Fig. 2c) is responsible for input/output operations with files from different sources. Specifically, it provides the ‘GenericFilePath’ class and memory-efficient generator (function) that can return (yield) ‘MWTabFile’ instances from different sources, e.g. single file, directory of files, archive of files on a local machine, URL address of the ‘mwTab’ formatted file, etc. Function (method) call diagram (see Fig. S1) shows how three modules ‘mwtab.py’, ‘tokenizer.py’, and ‘fileio.py’ work together during the ‘MWTabFile’ instance construction: the ‘fileio.read_files()’ method uses ‘fileio.GenericFilePath’ in order to determine what sources the ‘mwTab’ formatted file is coming from and then calls appropriate methods on the ‘mwtab.MWTabFile’ class in order to construct itself, i.e. top-level ‘mwtab.MWTabFile._build_mwtabfile’ and then ‘mwtab.MWTabFile._build_block’ in order to build each individual text block of the ‘mwTab’ formatted file into a usable ‘MWTabFile’ instance.

Since the ‘MWTabFile’ class is constructed using Python’s standard dictionary- and list-based data structures, the entire ‘MWTabFile’ instance can easily be serialized into an equivalent JSON representation. The ‘converter.py’ module (see Fig. 2d) is responsible for conversion between the JSONized representation of the ‘mwTab’ format and the regular ‘mwTab’ format. The ‘mwschema.py’ and ‘validator.py’ are two package modules designed to perform the validation of the ‘mwTab’ formatted files. The ‘mwschema.py’ provides the current schema definitions for the ‘mwTab’ format and the ‘validator.py’ module provide functions to validate individual text blocks as well as the entire ‘mwTab’ formatted file using those schema definitions. The schema definitions are implemented using the ‘schema’ Python library (“schema—validation just got Pythonic.”—Available at: https://github.com/keleshev/schema/). The ‘cli.py’ module provides a simple command-line interface that can be used to convert ‘mwTab’ formatted files to their JSON representation and back as well as validate files on the command-line. The command-line interface is implemented with the help of the ‘docopt’ Python library (“docopt—creates beautiful command-line interfaces.”—Available at: https://github.com/docopt/docopt).

The ‘__init__.py’ and ‘__main__.py’ (see Fig. 2a) are special Python specific modules (‘__init__.py’ marks ‘mwtab’ top-level directory as a Python package and ‘__main__.py’ specifies the top-level environment for the ‘mwtab’ package).

### Evaluation data

To evaluate the ‘mwtab’ package functionality and performance, we used all ‘mwTab’ formatted files available from Metabolomics Workbench Data Repository on August 30, 2017. Due to the fact that there was no easy way to download all ‘mwTab’ formatted files from the repository, we created a specialized Python script that downloads every single ‘mwTab’ formatted file using ‘STUDY_ID’.

### Evaluation of mwtab package

The ‘mwtab’ Python package is available within a version-controlled GitHub repository under a Berkeley Software Distribution 3-clause clear open source license (BSD 3-Clause Clear License). As a part of our development process, we implemented several unit tests for each module of the ‘mwtab’ package (see Fig. 2a) in order to validate functionality using the popular Python unit testing framework ‘pytest’ (“pytest unit testing framework.”—Available at: https://docs.pytest.org). We used the continuous integration service ‘Travis CI’ to build and test our ‘mwtab’ package against Python versions 2.7, and 3.4+, build information is available under the ‘mwtab’ package GitHub repo. In addition, we generated code test coverage reports that are also available under the GitHub repo (currently, tests cover 90% of the code base).

### The mwtab package documentation

Each function, class, and class method was documented using sphinx python documentation style, which allowed us to generate package API documentation directly from the source code. In addition, we wrote ‘User Guide’, ‘Tutorial’, and ‘API Reference’ documentation which is available under http://mwtab.readthedocs.io.

## Results

### JSON representation of the mwTab format

JavaScript object notation is an open standard file format commonly used for data-interchange on the web. Its advantages include human readability, widespread support for reading and writing by different programming languages (“JSON: JavaScript Object Notation.”—Available at: http://www.json.org/). It is built upon two main data structures: a collection of key-value pairs (i.e. equivalent to Python dictionary data structure) and an ordered collection of values (i.e. equivalent to Python list and array data structures). Because the main ‘mwtab’ package data representation layer (i.e. ‘MWTabFile’ class) is built upon standard Python dictionary and list data structures, ‘mwTab’ formatted files are easily serializable into their equivalent JSON representation. In other words, ‘MWTabFile’ class creates an interface for one-to-one mapping between Python nested dictionary- and list-based data structures and a JSONized representation of the ‘mwTab’ format. In addition, this design provides a very intuitive programming interface for access and manipulation of data and metadata stored in original ‘mwTab’ formatted files.

Figure S2 compares different text blocks in ‘mwTab’ format with their corresponding JSONized representation: text blocks containing “single key-single value” and multiline summary text blocks (Fig. S2a, b), specially formatted subject sample factors text blocks (Fig. S2c, d), text blocks containing MS experimental data on metabolites (Fig. S2e, f), and text block containing NMR experimental data on metabolites (Fig. S2g, h).

In comparison to the standard ‘mwTab’ format, the main advantages of the JSON representation are: (i) it enables easy access to data from other programming languages without implementing specific ‘mwTab’ parser for that language; and (ii) it enables faster reading/processing of the data stored in ‘mwTab’ formatted files due to highly optimized and efficient JSON parsers. Figures S5 and S6 show code examples for data access from JSONized ‘mwTab’ files using R with ‘jsonlite’ R library (Ooms 2014) and C++ with ‘JSON for Modern C++’ library (“JSON for Modern C++.”—Available at: https://github.com/nlohmann/json), respectively.

### The mwtab package interface

The ‘mwtab’ package can be used in several ways: (i) as a library within Python scripts for accessing and manipulating data and metadata stored in ‘mwTab’ formatted files; and (ii) as a command-line tool to convert between the ‘mwTab’ format and its equivalent JSONized representation as well as for data validation using predefined schema definitions for each of the text blocks and consistency checking.

To use ‘mwtab’ package as a library within Python scripts, first it is necessary to import it within a Python program or an interactive interpreter interface. Next, the ‘MWTabFile’ instance(s) can be created using the generator function ‘read_files’. This generator function instantiates ‘MWTabFile’ object(s) from many different file sources: a local file, a URL address of a file, ‘ANALYSIS ID’ of a file, directory and/or archive of multiple files. The generator function can be processed in several ways: for example, to process files one at a time by calling the Python ‘next()’ built-in function, to process every file in a for-loop, or to convert the generator into list of ‘MWTabFile’ instances. Once the ‘MWTabFile’ object is created, it can be utilized like any Python built-in dictionary- and list-based data structures, the data can be accessed and/or manipulated using keys (in case of dictionary) or indexes (in case of list). Table 1 summarizes common patterns for using ‘mwtab’ as a library, but more detailed examples are available under the ‘mwtab’ package tutorial.

**Table 1:** Common patterns for using the ‘mwtab’ as a library

Usage	Example
Reading	mwt_generator = mwtab.read_files(‘path_to_file’) mwtfile = next(mw_generator)
Access	mwtfile[‘PROJECT’][‘PROJECT_SUMMARY’]
Modification	mwtfile[‘PROJECT’][‘PROJECT_SUMMARY’] = ‘new project summary’
Printing	mwtfile.print_file(file_format=‘mwtab’)
	mwtfile.print_file(file_format=‘json’)
Writing	mwtfile.write(file_handle, file_format=‘mwtab’)
	mwtfile.write(file_handle, file_format=‘json’)

The ‘mwtab’ package also provides a simple command-line interface that can be used to validate and convert files from ‘mwTab’ format to its JSON representation and back. Figure S3 shows the current command-line interface.

Table 2 summarizes common patterns for using ‘mwtab’ as a command- line tool, but the ‘mwtab’ package tutorial documentation provides more detailed examples.

**Table 2:** Common patterns for using the ‘mwtab’ as a command-line tool

Command	Description	Example
convert	Convert between ‘mwTab’ and its JSON representation	$ python3 -m mwtab convert AN000001.txt AN000001.json \ --from_format=mwtab --to_format=json $ python3 -m mwtab convert AN000001.json AN000001.txt \ --from_format=json --to_format=mwtab
validate	Validate file(s)	$ python3 -m mwtab validate AN000001.txt $ python3 -m mwtab validate AN000001.json

### Data validation functionality of the mwtab package

The ‘mwtab’ Python package provides two modules designed to perform data validation: ‘validator.py’ and ‘mwschema.py’. Once the ‘mwTab’ formatted file is parsed into a ‘MWTabFile’ instance (object), the data can be validated against a predefined schema. The ‘mwschema.py’ module provides schema definitions based on the official ‘mwTab’ format specification for each text block of the ‘mwTab’ file. For example, Figure S4a shows an example for ‘#PROJECT’ text block from the ‘mwTab’ specification: it specifies that the ‘PROJECT_TITLE’, ‘PROJECT_SUMMARY’, ‘INSTITUTE’, ‘LAST_NAME’, ‘FIRST_NAME’, ‘ADDRESS’, ‘EMAIL’, and ‘PHONE’ fields are required, and ‘PROJECT_TYPE’, ‘DEPARTMENT’, ‘LABORATORY’, ‘FUNDING_SOURCE’, ‘PROJECT_COMMENTS’, ‘PUBLICATIONS’, ‘CONTRIBUTIONS’, and ‘DOI’ fields are optional. If a text block is missing a required filed, a descriptive error message will be raised during the validation process. Figure S4b shows an example of an error message that is generated due to missing the ‘PROJECT_TITLE’ required field. The type of value that is expected by the schema definition can also be provided. In addition to Python standard built-in types (e.g., ‘str’, ‘int’, ‘float’, etc.), regular expressions can be provided where appropriate to validate data, e.g. a regular expression can be passed as a value for ‘EMAIL’ and ‘PHONE’ fields to verify that they correspond to valid e-mail and phone formats. All schema definitions are provided within the ‘mwschema.py’ module in the GitHub repository and can be easily modified to strengthen the data validation functionality.

### The mwtab package performance

In order to test the performance of our ‘mwtab’ Python package, we downloaded every ‘mwTab’ formatted file and created simple Python script that imports the library, instantiates the ‘MWTabFile’ objects from directory of files one file at a time and reports how much time it took. Table 3 shows that that it took under 30 s to process files in both ‘mwTab’ and its JSON representation. Table 3 also shows that the JSON representation is more verbose and therefore occupies more disk space; however, its main benefit is in providing easy access to the data and metadata for other programming languages with JSON parsers but no ‘mwTab’ parser.

**Table 3:** The ‘mwtab’ package performance against ‘mwTab’ and its JSON representation formats

Format	‘mwTab’	JSON representation of ‘mwTab’
Number of files	634	634
Total size of files (MB)	290.1	2000
Time (s)	28	24

In order to reduce disk space usage, the entire directory can be converted and compressed into a single archive file. The ‘mwtab’ package provides facilities to read directly from zip, tar.gz, and tar.bz2 archives without requirement to decompress those files manually.

### Evaluating format and metadata in mwTab formatted files

**Fig. 3:**
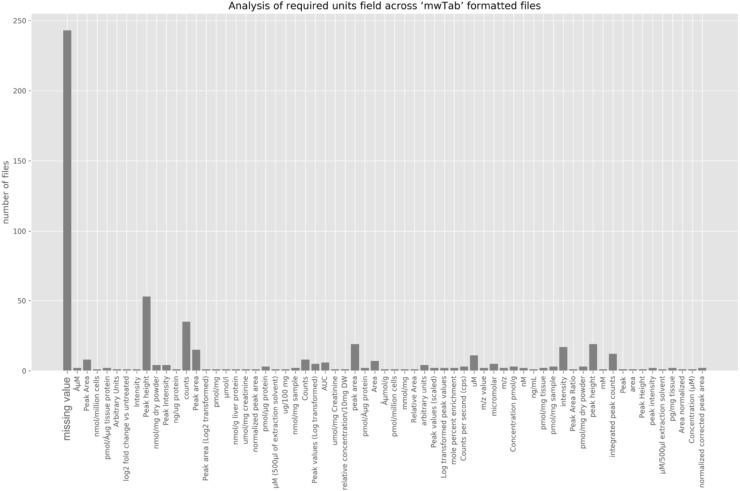
Analysis of required units field across ‘mwTab’ formatted files

During the development of ‘mwtab’ Python package, we were able to identify several inconsistencies between the official specification and the actual ‘mwTab’ formatted files provided by the Metabolomics Workbench at that time. For example, the ‘mwTab’ format specification says that each file has to start with ‘#METABOLOMICS WORKBENCH’ header string, but multiple files had single or multiple empty lines at the beginning of a file. Also, we were able to identify optional fields that were present within actual ‘mwTab’ formatted files but missing in the official specification. To demonstrate the type of exploratory analysis that can be performed using ‘mwtab’ package, we analyzed every ‘mwTab’ file in order to verify that mandatory data units fields are present and provide an actual units value. Figure 3 shows that 243 entries had missing value (the first bar on Fig. 3) for the required units field and some of the bars represent the same type units but split due to slight difference in their names (e.g. ‘Peak height’ vs. ‘peak height’, ‘Peak Intensity’ vs. ‘peak intensity’).

Additionally, we found that 302 were missing ‘#END’ statement that signals the end of the file, seven files had issues in their ‘#SUBJECT_SAMPLE_FACTORS’ text block, 20 files had issues in their ‘single key-single value’ pairs, and four files had some other minor formatting issues.

All of the ‘mwTab’ file format issues discovered with the help of ‘mwtab’ Python package were reported to Metabolomics Workbench and were promptly fixed within 1 week. The original files can be downloaded from a figshare repository along with the validation reports generated for each file. Current cleaned up files are available on the Metabolomics Workbench Data Repository.

## Conclusions

The ‘mwtab’ package is a useful Python library designed to provide facilities for parsing, accessing, and manipulating data stored in ‘mwTab’ and its JSONized equivalent representation. The JSONized representation provides several advantages to standard ‘mwTab’ format including improved reading speeds and enabling easy data access for other programming languages implementing JSON parser. Using internal Python and JSON data structures, ‘mwTab’ files can be validated with respect to consistency and completeness using specified schema definitions based on the official ‘mwTab’ format specification. The library has already proven useful in improving the quality of all ‘mwTab’ formatted files provided by the Metabolomics Workbench Data Repository, with respect to the official ‘mwTab’ format specification. The ‘mwtab’ package also provides an easy-to-use command-line interface designed to perform file conversion and file validation tasks. The ‘mwtab’ package provides extensive documentation, which includes a ‘User Guide’, ‘Tutorial’, and ‘API reference’ generated automatically from the source code and available on http://mwtab.readthedocs.io. The ‘mwtab’ package also includes automated unit-tests that perform testing of every module of the package as well as generates test coverage reports. We believe that the ‘mwtab’ package will help to improve metadata quality and data reusability of metabolomics data from Metabolomics Workbench Data Repository by downstream investigators through providing Python interfaces for data access and manipulation and through providing a JSONized representation of the ‘mwTab’ format for use in other programming languages.

## Electronic supplementary material

Below is the link to the electronic supplementary material.

Supplementary material 1 (DOCX 90 KB)

## Data Availability

Original data available at: https://figshare.com/s/8d5a837cdc3f500fbcaa.
