# Supplementary material for: A Python library for FAIRer access and deposition to the Metabolomics Workbench Data Repository
Source: Metabolomics. 2018 Apr 20;14(5):64. doi: 10.1007/s11306-018-1356-6 (PMC5910482; doi:10.1007/s11306-018-1356-6)
Supplement: Supplementary file 1 — Supplementary material 1 (DOCX 90 KB) [file 11306_2018_1356_MOESM1_ESM.docx]

# Supplemental Materials

| 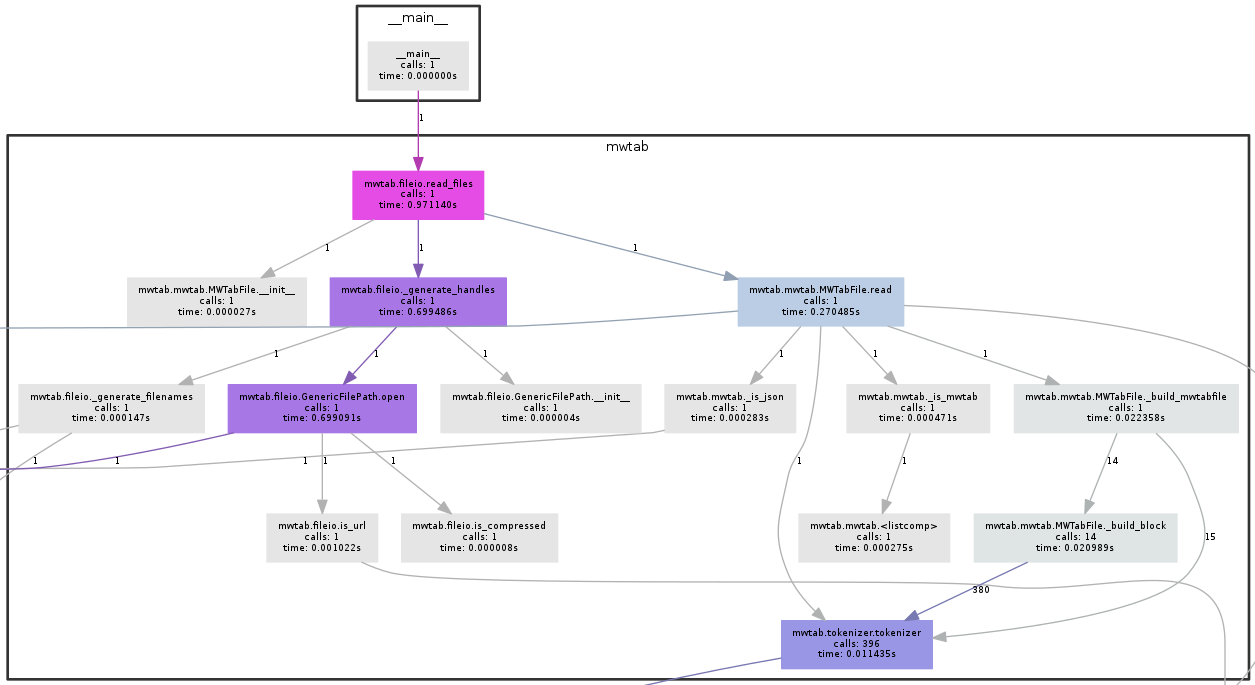 |
| --- |
| Figure S1. Function (method) call diagram during the creation of a single ‘MWTabFile’ instance (object). |

| **a** | #METABOLOMICS WORKBENCH STUDY_ID:ST000001 ANALYSIS_ID:AN000001  VERSION 1  CREATED_ON 2016-09-17  #PROJECT  PR:PROJECT_TITLE FatB Gene Project  PR:PROJECT_TYPE Genotype treatment  PR:PROJECT_SUMMARY Experiment to test the consequence of  PR:PROJECT_SUMMARY a mutation at the FatB gene (At1g08510)  PR:PROJECT_SUMMARY the wound-response of Arabidopsis  ... |
| --- | --- |
| **b** | {  "METABOLOMICS WORKBENCH": {  "STUDY_ID": "ST000001",  "ANALYSIS_ID": "AN000001",  "VERSION": "1",  "CREATED_ON": "2016-09-17"  },  "PROJECT": {  "PROJECT_TITLE": "FatB Gene Project",  "PROJECT_TYPE": "Genotype treatment",  "PROJECT_SUMMARY": "Experiment to test the consequence of\na mutation at the FatB gene (At1g08510)\nthe wound-response of Arabidopsis"  },  ...  } |
| **c** | #SUBJECT_SAMPLE_FACTORS: SUBJECT(optional)[tab]SAMPLE[tab]FACTORS(NAME:VALUE pairs separated by \|)[tab]Additional sample data  SUBJECT_SAMPLE_FACTORS - B212A02 Hours:0.5 \| Compactin:0 \| KLA:0  SUBJECT_SAMPLE_FACTORS - B219A02 Hours:0.5 \| Compactin:0 \| KLA:0  SUBJECT_SAMPLE_FACTORS - B226A02 Hours:0.5 \| Compactin:0 \| KLA:0  SUBJECT_SAMPLE_FACTORS - B212A03 Hours:0.5 \| Compactin:50uM \| KLA:0  SUBJECT_SAMPLE_FACTORS - B219A03 Hours:0.5 \| Compactin:50uM \| KLA:0  SUBJECT_SAMPLE_FACTORS - B226A03 Hours:0.5 \| Compactin:50uM \| KLA:0 |
| **d** | {  ...  "SUBJECT_SAMPLE_FACTORS": {  "SUBJECT_SAMPLE_FACTORS": [  {  "subject_type": "-",  "local_sample_id": "B212A02",  "factors": "Hours:0.5 \| Compactin:0 \| KLA:0",  "additional_sample_data": ""  },  ...  ...  } |
| **e** | #MS_METABOLITE_DATA  MS_METABOLITE_DATA:UNITS pmol/ug DNA  MS_METABOLITE_DATA_START  Samples...  Factors...  10Z-heptadecenoic acid 1.1200 0.4900 0.4400 0.4100 0.5400 0.5800  11_14_17-eicosatrienoic acid 4.4200 6.0000 3.2500 3.6100 7.7600 4.1900  11_14-eicosadienoic acid 0.1800 0.3600 0.1600 0.3100 0.4600 0.2500  ...  MS_METABOLITE_DATA_END |
| **f** | {  ...  "MS_METABOLITE_DATA": {  "MS_METABOLITE_DATA:UNITS": "pmol/ug DNA",  "MS_METABOLITE_DATA_START": {  "Samples": [...],  "Factors": [...],  "DATA": [  {"metabolite_name": "10Z-heptadecenoic acid",  "B212A02": "1.1200",  "B219A02": "0.4900",  "B226A02": "0.4400",  "B212A03": "0.4100",  "B219A03": "0.5400",  "B226A03": "0.5800"  },  ...  ]  }  },  ...  } |
| **g** | #NMR_BINNED_DATA  NMR_BINNED_DATA_START  Bin range(ppm) CDC029 CDC030 CDC032 CPL101 CPL102 CPL103  0.82...0.84 2.8253 3.1284 1.1065 1.5676 1.7775 1.7097  0.84...0.90 34.974 30.485 14.352 15.274 17.848 16.936  0.90...0.92 11.828 10.11 5.4776 7.1873 7.2929 8.0632  0.92...0.98 68.285 67.389 33.7 59.048 62.212 65.748  ...  NMR_BINNED_DATA_END |
| **h** | {  ...  "NMR_BINNED_DATA": {  "NMR_BINNED_DATA_START": {  "Fields": [  "Bin range(ppm)",  "CDC029",  "CDC030",  "CDC032",  "CPL101",  "CPL102",  "CPL103",  ],  "DATA": [  {"Bin range(ppm)": "0.82...0.84",  "CDC029": "2.8253",  "CDC030": "3.1284",  "CDC032": "1.1065",  "CPL101": "1.5676",  "CPL102": "1.7775",  "CPL103": "1.7097"  },  ...  ]  }  },  ...  } |
| Figure S2. Comparison between the ‘mwTab’ format and its internal Python dictionary-, list-based, JSON representation: a) Text blocks containing “single key-single value” and multiline summary blocks; b) JSON representation of ; c) subject sample factors text block; d) JSON representation of subject sample factors text block; e) text block with MS metabolite data; f) JSON representation of the MS data text block; g) text block with NMR data; h) JSON representation of the NRM data text block. | |

| The mwtab command-line interface  ~~~~~~~~~~~~~~~~~~~~~~~~~~~~~~~~  Usage:  mwtab -h \| --help  mwtab --version  mwtab convert (<from-path> <to-path>) [--from-format=<format>]  [--to-format=<format>]  [--validate] [--verbose]  mwtab validate <from-path> [--verbose]  Options:  -h, --help Show this screen.  --version Show version.  --verbose Print what files are processing.  --validate Validate the mwTab file.  --from-format=<format> Input file format, available formats: mwtab, json [default: mwtab].  --to-format=<format> Output file format, available formats: mwtab, json [default: json].  --mw-rest=<url> URL to MW REST interface. |
| --- |
| Figure S3. The ‘mwtab’ package command-line interface. |

| **a** | project_schema = Schema(  {  "PROJECT_TITLE": str,  Optional("PROJECT_TYPE"): str,  "PROJECT_SUMMARY": str,  "INSTITUTE": str,  Optional("DEPARTMENT"): str,  Optional("LABORATORY"): str,  "LAST_NAME": str,  "FIRST_NAME": str,  "ADDRESS": str,  "EMAIL": str,  "PHONE": str,  Optional("FUNDING_SOURCE"): str,  Optional("PROJECT_COMMENTS"): str,  Optional("PUBLICATIONS"): str,  Optional("CONTRIBUTORS"): str,  Optional("DOI"): str  }  ) |
| --- | --- |
| **b** | Traceback:  File "mwtab/mwtab/validator.py", line 82, in validate_file  section = validate_section(mwtabfile, section_key, section_schema_mapping)  File "mwtab/mwtab/validator.py", line 56, in validate_section  validated = schema.validate(mwtabfile[section_key])  File "/usr/lib/python3/site-packages/schema.py", line 265, in validate  **SchemaMissingKeyError('Missing keys: ' + s_missing_keys, e)**  **schema.SchemaMissingKeyError: Missing keys: 'PROJECT_TITLE'** |
| Figure S4. Example of the schema definition for ‘#PROJECT’ text block. | |

| **R example using ‘jsonlite’ library** |
| --- |
| > **# load library**  > library(jsonlite)  >  > **# load JSONized mwTab file**  > mwtfile <- fromJSON("ST000001_AN000001.json")  >  > **# print text block names (top-level keys)**  > names(mwfile)  [1] "METABOLOMICS WORKBENCH"  [2] "PROJECT"  [3] "STUDY"  [4] "SUBJECT"  [5] "SUBJECT_SAMPLE_FACTORS"  [6] "COLLECTION"  [7] "TREATMENT"  [8] "SAMPLEPREP"  [9] "CHROMATOGRAPHY"  [10] "ANALYSIS"  [11] "MS"  [12] "MS_METABOLITE_DATA"  [13] "METABOLITES"  >  > **# access key-value data**  > mwtfile$PROJECT$PROJECT_TITLE  [1] "FatB Gene Project"  > mwtfile$PROJECT$PROJECT_SUMMARY  [1] "Experiment to test the consequence of a mutation at the FatB gene (At1g08510)\nthe wound-response of Arabidopsis"  >  > **# access subject sample factors text block**  > mwtfile$SUBJECT_SAMPLE_FACTORS$SUBJECT_SAMPLE_FACTORS$factors  [1] "Arabidopsis Genotype:Wassilewskija (Ws) \| Plant Wounding Treatment:Control - Non-Wounded"  [2] "Arabidopsis Genotype:Wassilewskija (Ws) \| Plant Wounding Treatment:Control - Non-Wounded"  [3] "Arabidopsis Genotype:Wassilewskija (Ws) \| Plant Wounding Treatment:Control - Non-Wounded"  [4] "Arabidopsis Genotype:Wassilewskija (Ws) \| Plant Wounding Treatment:Control - Non-Wounded"  [5] "Arabidopsis Genotype:Wassilewskija (Ws) \| Plant Wounding Treatment:Control - Non-Wounded"  [6] "Arabidopsis Genotype:Wassilewskija (Ws) \| Plant Wounding Treatment:Control - Non-Wounded"  [7] "Arabidopsis Genotype:Wassilewskija (Ws) \| Plant Wounding Treatment:Wounded"  [8] "Arabidopsis Genotype:Wassilewskija (Ws) \| Plant Wounding Treatment:Wounded"  [9] "Arabidopsis Genotype:Wassilewskija (Ws) \| Plant Wounding Treatment:Wounded"  [10] "Arabidopsis Genotype:Wassilewskija (Ws) \| Plant Wounding Treatment:Wounded"  [11] "Arabidopsis Genotype:Wassilewskija (Ws) \| Plant Wounding Treatment:Wounded"  [12] "Arabidopsis Genotype:Wassilewskija (Ws) \| Plant Wounding Treatment:Wounded"  >  **> # access MS metabolite data text block**  > mwtfile$MS_METABOLITE_DATA$MS_METABOLITE_DATA_START$DATA  metabolite_name LabF_115904 LabF_115909  1 1,2,4-benzenetriol 1874 3566  2 1-monostearin 987 450  3 2-hydroxyvaleric acid 771 931  4 3-phosphoglycerate 2039 2005  5 5-hydroxynorvaline NIST 2297 2403  6 adenosine 538 407  7 adenosine-5-monophosphate 2153 1650  8 adipic acid 2197 8148  9 agmatine 2334 2259  10 alanine 122481 121790 |
| Figure S5. Code example demonstrating access to different text blocks within R script using ‘jsonlite’ library [11]. |

| **C++ example using ‘JSON for modern C++’ library** |
| --- |
| #include <iostream>  #include <fstream>  #include "json.hpp"  // for convenience  using json = nlohmann::json;  int main() {  json mwtfile;  std::ifstream is("ST000001_AN000001.json");  is >> mwtfile;  // print text block names (top-level keys)  for (auto it = mwtfile.begin(); it != mwtfile.end(); ++it)  std::cout << it.key() << "\n";  // access key-value data  std::cout << mwtfile["PROJECT"]["PROJECT_TITLE"] << "\n";  std::cout << mwtfile["PROJECT"]["PROJECT_SUMMARY"] << "\n";  // access subject sample factors text block  for (int i = 0; i < mwfile["SUBJECT_SAMPLE_FACTORS"]["SUBJECT_SAMPLE_FACTORS"].size(); ++i)  std::cout << mwtfile["SUBJECT_SAMPLE_FACTORS"]["SUBJECT_SAMPLE_FACTORS"][i]["factors"] << "\n";  // access MS metabolite data text block  std::cout << mwtfile["MS_METABOLITE_DATA"]["MS_METABOLITE_DATA_START"]["DATA"] << "\n";  }  // Output:  ANALYSIS  CHROMATOGRAPHY  COLLECTION  METABOLITES  METABOLOMICS WORKBENCH  MS  MS_METABOLITE_DATA  PROJECT  SAMPLEPREP  STUDY  SUBJECT  SUBJECT_SAMPLE_FACTORS  TREATMENT  "FatB Gene Project"  "Experiment to test the consequence of a mutation at the FatB gene (At1g08510)\nthe wound-response of Arabidopsis"  "Arabidopsis Genotype:Wassilewskija (Ws) \| Plant Wounding Treatment:Control - Non-Wounded"  "Arabidopsis Genotype:Wassilewskija (Ws) \| Plant Wounding Treatment:Control - Non-Wounded"  "Arabidopsis Genotype:Wassilewskija (Ws) \| Plant Wounding Treatment:Control - Non-Wounded"  "Arabidopsis Genotype:Wassilewskija (Ws) \| Plant Wounding Treatment:Control - Non-Wounded"  "Arabidopsis Genotype:Wassilewskija (Ws) \| Plant Wounding Treatment:Control - Non-Wounded"  "Arabidopsis Genotype:Wassilewskija (Ws) \| Plant Wounding Treatment:Control - Non-Wounded"  "Arabidopsis Genotype:Wassilewskija (Ws) \| Plant Wounding Treatment:Wounded"  "Arabidopsis Genotype:Wassilewskija (Ws) \| Plant Wounding Treatment:Wounded"  "Arabidopsis Genotype:Wassilewskija (Ws) \| Plant Wounding Treatment:Wounded"  "Arabidopsis Genotype:Wassilewskija (Ws) \| Plant Wounding Treatment:Wounded"  "Arabidopsis Genotype:Wassilewskija (Ws) \| Plant Wounding Treatment:Wounded"  "Arabidopsis Genotype:Wassilewskija (Ws) \| Plant Wounding Treatment:Wounded"  {"metabolite_name":"1,2,4-benzenetriol","LabF_115904":"1874","LabF_115909":"3566"}  {"metabolite_name":"1-monostearin","LabF_115904":"987","LabF_115909":"450"}  {"metabolite_name":"2-hydroxyvaleric acid","LabF_115904":"771","LabF_115909":"931"}  {"metabolite_name":"3-phosphoglycerate","LabF_115904":"2039","LabF_115909":"2005"}  {"metabolite_name":"5-hydroxynorvaline NIST","LabF_115904":"2297","LabF_115909":"2403"}  {"metabolite_name":"adenosine","LabF_115904":"538","LabF_115909":"407"}  {"metabolite_name":"adenosine-5-monophosphate","LabF_115904":"2153","LabF_115909":"1650"}  {"metabolite_name":"adipic acid","LabF_115904":"2197","LabF_115909":"8148"}  {"metabolite_name":"agmatine","LabF_115904":"2334","LabF_115909":"2259"}  {"metabolite_name":"alanine","LabF_115904":"122481","LabF_115909":"121790"} |
| Figure S6. Code example demonstrating access to different text blocks within C++ program using ‘JSON for Modern C++’ library [12]. |
